# Evaluation of the therapeutic efficacy of the dextromethorphan–bupropion combination and its mediating pathways in a mouse model of major depressive disorder

**DOI:** 10.3389/fphar.2026.1910199

**Published:** 2026-08-19

**Authors:** İlay Buran Kavuran, Ebru Önalan, Zeliha İrem Türk, Hatice Eröksüz

**Affiliations:** 1 Department of Medical Biology, Faculty of Medicine, Fırat University, Elazığ, Türkiye; 2 Department of Veterinary Pathology, Faculty of Veterinary Medicine, Fırat University, Elazığ, Türkiye

**Keywords:** caspase-3, dextromethorphan-bupropion, major depressive disorder, NMDA, TRPM2

## Abstract

**Background:**

Major depressive disorder (MDD) is associated with multiple pathophysiological mechanisms, including dysregulation of monoaminergic systems, excessive glutamatergic activity, reduced neurotrophic support, inflammation, and oxidative stress. The combination of dextromethorphan, an N-methyl-D-aspartate (NMDA) receptor antagonist and sigma-1 receptor agonist, with bupropion, a dopamine/norepinephrine reuptake inhibitor, has been reported to exert rapid antidepressant effects. In the present study, we evaluated the therapeutic effects of the dextromethorphan–bupropion combination on behavioral outcomes and neurotransmitter, inflammatory, and apoptotic pathways in a mouse model of MDD.

**Methods:**

A total of 36 BALB/c mice were randomly assigned to four groups: Control, CUMS, DXM + BUP, and CUMS + DXM + BUP. The chronic unpredictable mild stress (CUMS) protocol was applied for 6 weeks. Depressive-like behaviors were assessed using the sucrose preference test, open field test, and forced swim test. At the end of the experiment, hippocampal and prefrontal cortex tissues were collected. Serum serotonin, dopamine, GABA, glutamate, and norepinephrine levels were quantified by ELISA. Gene expression levels of *Slc6a15*, *Bdnf*, *Ntrk2*, *Gdnf*, *Gfra1*, *Ngf*, *Ntf3*, *Ntf4*, *Creb1*, *Trpm2*, *Parp1*, *Parg*, *Casp3*, *Mapk14*, *Mapk1*, *Nfkb1*, *Tnf*, and *Il6* were analyzed by qRT-PCR. GluN2B, TRPM2, and Caspase-3 immunoreactivity was evaluated by immunohistochemistry.

**Results:**

The CUMS group exhibited significant weight loss, anhedonia, increased immobility, elevated glutamate and norepinephrine levels, and reduced serotonin and dopamine levels. In addition, decreased expression of neurotrophic genes and increased expression of *Trpm2*, *Mapk14*, *Tnf*, and *Il6*, together with elevated Caspase-3 immunoreactivity, was observed. Administration of the dextromethorphan–bupropion combination significantly reversed these behavioral and molecular alterations. Immunohistochemical analyses further demonstrated reduced TRPM2, GluN2B, and Caspase-3 immunoreactivity in the treatment groups compared with the CUMS group.

**Conclusion:**

The dextromethorphan–bupropion combination ameliorated CUMS-induced depressive phenotypes at behavioral, neurotrophic, inflammatory, and apoptotic levels. These findings suggest that this combination represents a potent multimodal therapeutic candidate for stress-related depression.

## 1 Introduction

Major depressive disorder (MDD) is a highly prevalent psychiatric condition associated with persistent depressive symptoms and accompanying alterations in brain structure and function (Malhi and Mann, 2018). The pharmacological actions of currently available antidepressants primarily target receptors and enzymes involved in the monoamine hypothesis of depression, aiming to enhance synaptic signaling of three key monoamines: dopamine (DA), serotonin (5-HT), and noradrenaline (NOR) Beyond monoaminergic pathways, growing evidence suggests that additional neurotransmitter systems, including acetylcholine (ACh), gamma-aminobutyric acid (GABA), glutamate (Glu), and their respective receptors, play important roles in the pathophysiology of MDD (Sanacora et al., 2012). The etiology of major depression involves not only altered monoamine levels but also changes in receptor density and sensitivity, gene expression profiles, and downstream molecular signaling cascades initiated by these receptors (Stahl, 2026; Castrén and Monteggia, 2021).

Recent studies suggest that MDD is associated not only with disruptions in synaptic neurochemical activity but also with structural alterations and degeneration in key central regions such as the hippocampus. Chronic stress–related overactivation of the hypothalamic–pituitary–adrenal (HPA) axis is thought to promote MDD by altering hippocampal synaptic plasticity regulators, including brain-derived neurotrophic factor (BDNF), glial cell line–derived neurotrophic factor (GDNF), nerve growth factor (NGF), neurotrophin-3/4, and cAMP response element-binding protein (CREB) (Castrén and Monteggia, 2021). Moreover, stress-related HPA axis overactivation enhances glutamatergic activity, leading to elevated glucocorticoid levels, which are believed to underlie neuroanatomical alterations within the hippocampus (Sanacora et al., 2012) The neutral amino acid transporter SLC6A15, expressed in neurons, has been proposed as a candidate gene conferring vulnerability to major depression and stress, given its role in regulating hippocampal glutamate content. Reduced SLC6A15 expression has been associated with reduced availability of glutamate and glutamine in hippocampal tissue, whereas *Slc6a15* overexpression increases glutamate/glutamine concentrations. Decreased *Slc6a15* levels have been reported to induce alterations in glutamate volume and HPA axis activity, thereby contributing to depressive symptomatology (Santarelli et al., 2017). Moreover, prolonged stress enhances glutamatergic activity, which may promote neuronal loss through overactivation of N-methyl-D-aspartate receptors (NMDAR). Evidence from both preclinical and clinical studies indicates that NMDAR blockade attenuates stress-related neurotoxicity and produces antidepressant-like outcomes, partly through modulation of serotonergic and dopaminergic signaling (Sanacora et al., 2012; Hashimoto, 2011). In this context, NMDA antagonists have attracted increasing interest in antidepressant research. Among these agents, the dextromethorphan/bupropion (DXM/BUP) combination, which integrates the NMDA receptor antagonist and sigma-1 receptor agonist dextromethorphan with the dopamine/noradrenaline reuptake inhibitor bupropion, has emerged as a promising therapeutic strategy (Stahl, 2019; Nguyen and Matsumoto, 2015). Whereas conventional monoaminergic antidepressants typically require 4–8 weeks to achieve clinically meaningful responses or remission, DXM/BUP has been reported to produce antidepressant effects within approximately 1 week of treatment (Iosifescu, 2022a; Stahl, 2013; Tabuteau, 2022a). The primary antidepressant potential of dextromethorphan is thought to derive from its non-competitive antagonism of NMDA receptors in the central nervous system. Bupropion competitively inhibits CYP2D6, prolonging the half-life of dextromethorphan to approximately 22 h, thereby maintaining therapeutic plasma levels and ensuring sustained NMDA receptor antagonism. Beyond NMDA blockade, dextromethorphan also acts as a sigma-1 receptor agonist and inhibits serotonin and norepinephrine reuptake (McCarthy et al., 2023; Khabir et al., 2022; Lepkowsky, 2022). Sigma-1 receptors modulate voltage and ligand-gated ion channels, while NMDA receptor activation by glutamate promotes Ca^2+^ influx into postsynaptic neurons, contributing to synaptic plasticity (Sanacora et al., 2012). Accordingly, one potential mediator of the therapeutic effects of the DXM/BUP combination may involve the Ca^2+^-permeable transient receptor potential melastatin 2 (TRPM2) channel and its associated signaling pathways. Activation of NMDA receptors and *Trpm2* channels has been shown to induce mitochondrial reactive oxygen species (mROS) production and apoptosis in neurons, thereby contributing to depressive pathology (Yıldızhan and Nazıroğlu, 2023). Animal studies have demonstrated that chronic mild stress markedly increases brain reactive oxygen species (ROS) levels and promotes MDD development through upregulation of the PARP-1/TRPM2 pathway (Yıldızhan and Nazıroğlu, 2023; Wang et al., 2023; Blenn et al., 2011). Loss of *Trpm2* channels has also been reported to alter NMDA receptor mediated synaptic plasticity, particularly long-term depression (Xie et al., 2011). Furthermore, ROS generated under chronic stress conditions play a substantial role in neuroinflammation, a process strongly implicated in depression. NMDA receptors have been shown to contribute to inflammation-related depressive states (Francija et al., 2019), while *Trpm2* modulates NMDA receptor expression (Alim et al., 2013). ROS-triggered Ca^2+^ entry through *Trpm2* leads to activation of p38, ERK, JNK (MAPK), and NF-κB pathways, thereby modulating the production of pro-inflammatory cytokines *(Tnf-Α, Il-6*) (Malko et al., 2019). Despite these advances, preclinical studies investigating the molecular mechanisms underlying the antidepressant effects of the dextromethorphan–bupropion combination, particularly its relationship with *Trpm2* channels and NMDA receptor signaling in MDD, remain limited. Therefore, in the present study, we aimed to investigate the therapeutic efficacy of the dextromethorphan–bupropion combination in a mouse model of MDD by examining neurotransmitter networks, neurotrophic factors, behavioral outcomes, the ROS-mediated PARP1–PARG–TRPM2–Caspase-3 axis, and the TRPM2-associated p38–ERK–NF-κB–TNF-α/IL-6 inflammatory pathway.

## 2 Materials and methods

### 2.1 Experimental design

**FIGURE 1:**
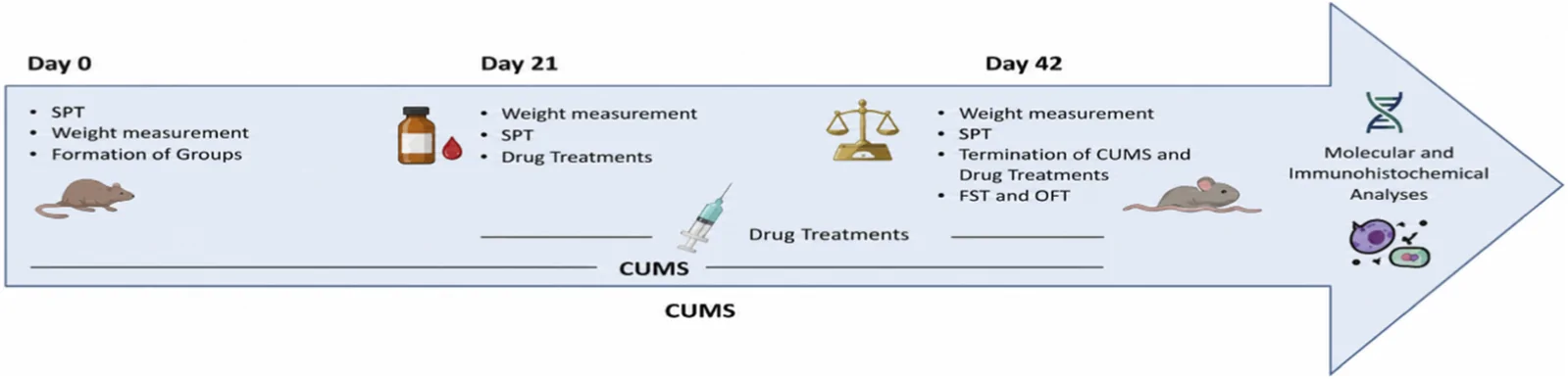
Study Design, SPT, Sucrose preference test; FST, Forced swimming test; OFT, Open field test; CUMS, Chronic unpredictable mild stress.

Thirty-six adult male BALB/c albino mice (2–3 months old) were included in the study. Sample size was determined by power analysis (α = 0.05, 1−β = 0.95, effect size = 1.66), indicating a minimum of nine animals per group. Mice were housed in polycarbonate cages (50 × 30 × 20 cm) under controlled conditions (≈23 °C; 12h light/dark cycle) with free access to food and water. Animals were assigned to four groups: Control, CUMS, DXM + BUP, and CUMS + DXM + BUP. While the Control group remained under standard housing, the remaining groups were subjected to experimental procedures as outlined in the CUMS protocol (Figure 1). Only male mice were included to minimize biological variability associated with the estrous cycle and hormonal fluctuations, which may influence behavioral, neurochemical, and molecular responses to chronic stress and antidepressant treatment. Therefore, sex was not included as an experimental factor in the study design or statistical analyses. The study was conducted following ethical authorization from the animal experiments ethics committee (Fırat University; 2024/07-12).

### 2.2 Chronic unpredictable mild stress

Chronic unpredictable mild stress remains one of the most frequently used and scientifically validated methods in experimental models of MDD. In the present study, a protocol was developed based on Willner’s CUMS model (Willner, 2005). To induce CUMS, mice were randomly exposed to 2 or 3 stressors per day, as specified in Table 1, for a total duration of 6 weeks. Body weight was recorded with a precision balance on Days 0, 21, and 42 of the CUMS protocol.

**TABLE 1:** Chronic unpredictable mild stress protocol.

Stressor	Description	Duration	Schedule
Overnight light exposure	Continuous illumination during the dark phase	12 h	Monday, Saturday
Food deprivation	Food removed	12–24 h	Tuesday, Friday
Water deprivation	Water deprivation followed by 30 min exposure to an empty water bottle	18–24 h	Monday, Wednesday
Moist bedding	Housing with damp bedding	24 h	Wednesday
Bedding deprivation	Housing without wood-chip bedding	24 h	Thursday, Saturday
Social isolation	Individual housing in a separate cage	24 h	Tuesday, Sunday
Novel object exposure	Placement of a novel object in the home cage	24 h	Thursday, Sunday
Cage tilt	Cage inclined at a 45° angle	8 h	Tuesday, Friday
White noise	80 dB white noise exposure	1 h	Monday, Thursday, Sunday
Strobe light	Flashing light exposure	3–4 h	Wednesday, Friday, Sunday
Daytime darkness	Complete darkness during the normal light phase	3 h	Monday, Thursday

To induce the chronic unpredictable mild stress (CUMS) model, mice assigned to the stress groups were exposed to a randomized sequence of mild stressors over the experimental period (Table 1). The stressors included overnight light exposure (12 h), daytime dark (3 h), food deprivation (12–24 h), water deprivation (18–24 h) followed by 30 min exposure to an empty water bottle, damp bedding (24 h), bedding deprivation (24 h), individual housing (24 h), placement of a novel object in the home cage (24 h), cage tilting at a 45° angle (8 h), white noise exposure (80 dB, 1 h), and flashing light exposure (3–4 h). The order, duration, and timing of the stressors were varied throughout the protocol to maintain unpredictability and minimize habituation to repeated stress exposure. Abbreviations: Mon, Monday; Tue, Tuesday; Wed, Wednesday; Thu, Thursday; Fri, Friday; Sat, Saturday; Sun, Sunday.

### 2.3 Drugs

Before the main experiment, a preliminary dose-finding study was conducted to determine an appropriate treatment dose for the DXM + BUP combination, as no well-established dosing protocol for this combination in the CUMS mouse model was available. Two total combination doses (0.15 and 0.30 mg/kg) were evaluated in separate groups of five mice each under the same experimental conditions as the main study. Behavioral responses and general tolerability were assessed throughout the treatment period. The 0.30 mg/kg total combination dose consistently produced a more pronounced antidepressant-like effect without observable adverse effects and was therefore selected for the main experiment. In clinical practice, the fixed-dose formulation contains 45 mg dextromethorphan and 105 mg bupropion (Iosifescu, 2022a). In the present study, the original 3:7 (DXM:BUP) ratio of the clinical formulation was preserved to maintain the pharmacological relationship between the two compounds, rather than directly converting the absolute human dose. Accordingly, the treatment consisted of 0.09 mg/kg dextromethorphan and 0.21 mg/kg bupropion (total combination dose: 0.30 mg/kg). The DXM + BUP combination was dissolved in physiological saline and administered once daily by oral gavage during the final 21 consecutive days of the 42-day CUMS protocol to mice in the DXM + BUP and CUMS + DXM + BUP groups. Behavioral assessments were performed 4 h after the final drug administration (McCarthy et al., 2023; Li et al., 2016).

### 2.4 Behavioral assessment of depression-like behaviors

#### 2.4.1 Sucrose preference test

To verify successful establishment of the depressive phenotype, sucrose preference testing (SPT) was conducted in all groups on Days 0, 21, and 42 of the CUMS protocol. A sucrose preference below 65% was considered indicative of anhedonia (Strekalova et al., 2023). The SPT was performed at the beginning (Day 0), midpoint (Day 21), and end (Day 42) of the CUMS protocol. For the test, mice were habituated to two bottles of 1% sucrose solution for 24 h, followed by replacement of one bottle with water. After 15 h, fluid intake was recorded and sucrose preference was calculated as: sucrose intake/ (total fluid intake) × 100. Tests were initiated during the active phase without prior food or water deprivation. In addition to SPT, depressive-like behaviors were evaluated using the forced swim test (FST) and open field test (OFT), with OFT performed first to avoid stress-related interference. All behavioral assessments were carried out 4 h after the final drug administration (Li et al., 2016).

#### 2.4.2 Open field test

On the final day of the experiment, the OFT was conducted using an open-top Plexiglas arena divided into 25 equal squares (100 × 100 × 40 cm). Each mouse was placed in a corner of the arena and allowed to freely explore. Locomotor activity was quantified as the number of squares crossed within 5 min, and exploratory activity was assessed by counting the number of rearing events (standing on the hind limbs) during the same period. Fear/anxiety-related behavior was evaluated by the number of fecal boli produced during the test. An increase in locomotor and exploratory activity together with a decrease in defecation was interpreted as reduced emotionality, whereas decreased locomotor and exploratory activity with increased defecation was interpreted as increased emotionality (i.e., anxiety). After each animal, the apparatus was cleaned with 70% alcohol (Li et al., 2016).

#### 2.4.3 Forced swimming test

One hour after completion of the OFT, the FST was performed to evaluate depressive-like behaviors. Procedures were based on the protocol described by Porsolt and colleagues (Porsolt et al., 1977). A cylindrical Plexiglas container (27 cm diameter, 50 cm height) was filled with water (24 °C–26 °C) to a depth of 30 cm. On the first day, mice underwent a 15-min swimming session. On the following day, mice were subjected to a 6-min forced swim session. Video recordings were obtained throughout the session to quantify immobility (periods during which the body remained motionless with the head above water), swimming, and climbing behaviors. The final 5 min of the session were scored by a blinded observer at 5-s intervals for immobility, swimming, and climbing (Porsolt et al., 1977).

After completion of the behavioral assessments, animals were sacrificed and brain tissues were rapidly collected. Following dissection, hippocampal and prefrontal cortex samples were rapidly cryopreserved in liquid nitrogen and maintained at −80 °C. Blood samples were centrifuged (5,000 × g, 5 min), and serum was preserved at −80 °C. All samples were subsequently used for molecular analyses.

### 2.5 Molecular analyses

#### 2.5.1 Enzyme linked immunosorbent assay (ELISA)

ELISA was used to quantify serum levels of serotonin, dopamine, glutamate, GABA, acetylcholine, and norepinephrine (Reed Biotech LTD., China; Cat. Nos. RE10166, RE10135, RE10060, RE10172, RE10129, and RE10132, respectively). All assays were performed strictly according to the manufacturer’s instructions and specifications.

#### 2.5.2 Quantitative real-time polymerase chain reaction (qRT-PCR)

Real-time PCR analyses were performed to determine the expression levels of *Slc6a15*, *Bdnf*, *Ntrk2*, *Gdnf*, *Gfra1*, *Ngf*, *Ntf3*, *Ntf4*, *Creb1*, *Trpm2*, *Parp1*, *Parg*, *Casp3*, *Mapk14* (p38), *Mapk1* (Erk), *Nfkb1*, *Tnf*, and *Il6*. Hippocampal and prefrontal cortex tissues were removed from −80 °C storage and equilibrated to room temperature, followed by RNA isolation using the standard TRIzol protocol. RNA concentration was quantified by measuring absorbance at 260 nm using spectrophotometry. Complementary DNA (cDNA) was generated with the High-Capacity cDNA Reverse Transcription Kit (Applied Biosystems, USA; Cat. No. 4368814) in a Veriti Thermal Cycler (Applied Biosystems, USA). Gene-specific primer sequences were designed using the Qiagen GeneGlobe platform (Qiagen, Hilden, Germany) based on the corresponding NCBI reference transcript sequences. The primer sequences used in this study are presented in Table 2. Target gene amplification was carried out using SYBR Green–based gene-specific primers (Sentebiolab, Ankara, Türkiye; Table 2). For q-PCR, reaction mixtures were prepared using cDNA samples together with iTaq Universal SYBR Green Supermix (Bio-Rad, USA; Cat. No. 172-5121). All reactions were performed in technical duplicates, and no-template controls were included to monitor potential contamination. Amplification and detection of target gene expression were carried out using an Applied Biosystems 7500 Real-Time PCR System (Applied Biosystems, Singapore). GAPDH was used as the housekeeping gene (Qiagen, USA; Cat. No. QT00079247). Fold changes in target gene expression were quantified using the 2^−ΔΔCT^ normalization method.

**TABLE 2:** Genes and primer sequences analyzed by RT-PCR.

Gene name	NCBI mRNA code	Forward	Reverse
Mmu-Slc6a15	NM_001252330.1	5'GAG​TCT​TCG​TTG​GAC​AGG​AGG3'	5'GAA​GCA​CCA​GAG​GGT​GTC​TTT​A3'
Mmu-TRPM2	NM_001411900.1	5'CTT​CTC​CAT​CCT​GGT​GCG​AGT​A3'	5'CGC​CAT​CTT​CAT​TAG​TCC​ACA​GC3'
Mmu-PARP1	NM_007415.3	5'CTC​TCC​CAG​AAC​AAG​GAC​GAA​G3'	5'CCG​CTT​TCA​CTT​CCT​CCA​TCT​TC3'
Mmu-PARG	NM_001359915.1	5'GAA​GAA​GTG​GCT​GGG​AAC​TCC​T3'	5'GTT​TCG​GAA​CCT​CTC​CTG​CTC​T3'
Mmu-BDNF	NM_001048139.1	5′-GGT​ATC​CAA​AGG​CCA​ACT​GA-3′	5′-CTT​ATG​AAT​CGC​CAG​CCA​AT-3′
Mmu-GDNF	NM_001301332.1	5'CCT​TCG​CGC​TGA​CCA​GTG​ACT3'	5'GCC​GCT​TGT​TTA​TCT​GGT​GAC​C3'
Mmu-TrkB	NM_001025074.3	5′-CGC​CCT​GTG​AGC​TGA​ACT​CTG-3′	5′-CTG​CTT​CTC​AGC​TGC​CTG​ACC-3′
Mmu-GFRα1	NM_001285457.3	5'TGT​CTT​TCT​GAT​AAT​GAT​TAC​GGA3'	5'CTA​CGA​TGT​TTC​TGC​CAA​TGA​TA 3'
Mmu-NT-3	NM_001164034.2	5'-CTC​ATT​ATC​AAG​TTG​ATC​CA	5'-CCT​CCG​TGG​TGA​TGT​TCT​ATT3'
Mmu-NT-4/5	NM_001164034.2	5'-CCC​TGC​GTC​AGT​ACT​TCT​TCG​AGA​C	5'-CTG​GAC​GTC​AGG​CAC​GGC​CTG​TTC
Mmu-CREB1	NM_001037726.1	5′ AGC​TGG​CCT​GTC​CCA​CTG​CT3′	5′ ACC​ATT​CTG​AAC​ACA​AAG​CAG​CCA3′
Mmu-Caspase-3	NM_001284409.1	5'AGC​AGC​TTT​GTG​TGT​GTG​ATT​CTA​A3'	5'AGT​TTC​GGC​TTT​CCA​GTC​AGA​C3'
Mmu-p38 (MAPK-14)	NM_001168508.1	5′-TGG​AGA​AGA​TGC​TCG​TTT​TG-3	5′-GTG​GCA​CAA​AGC​TGA​TGA​CT-3′
Mmu-ERK (MAPK-1)	NM_001038663.1	5′-GAG​CTG​TGC​AGC​AAA​CAG​TC-3′	5′-CTT​CCG​GAA​GCG​TGA​TAG​G-3′
Mmu-NF-Kb	NM_001410442.1	5'AGA​GAA​GCA​CAG​ATA​CCA​CTA​AG3'	5'CAG​CCT​CAT​AGA​AGC​CAT​CC3'
Mmu-TNF-α	NM_001278601.1	5'-CGA​GTG​ACA​AGC​CTG​TAG​CCC-3'	5'-GTC​TTT​GAG​ATC​CAT​GCC​GTT​G-3'
Mmu-IL-6	NM_001314054.1	5′-ACA​ACC​ACG​GCC​TTC​CCT​AC-3	5′-TCT​CAT​TTC​CAC​GAT​TTC​CCA​G-3′

#### 2.5.3 Immunohistochemical analyses

Immunohistochemical evaluations were performed on brain tissues for Caspase-3, GluN2B, and TRPM2. Following decapitation, brains were rapidly removed, fixed in 10% formaldehyde, processed through routine tissue tracking procedures, and embedded in paraffin blocks for histochemical examination. Sections (5–6 µm) were obtained and mounted on poly-L-lysine–coated slides. Immunohistochemical detection of *Caspase-3*, *GluN2B*, and *TRPM2* was carried out using the streptavidin–biotin peroxidase complex technique. After deparaffinization in xylene and rehydration through graded alcohols, sections were subjected to antigen retrieval by boiling in citrate buffer. Following endogenous peroxidase blocking and protein blocking, sections were incubated with primary antibodies for 1 h at room temperature. Subsequently, sections were incubated sequentially with biotinylated secondary antibody and streptavidin–peroxidase. Diaminobenzidine (DAB) chromogen was then applied to develop staining under light microscopy. Sections were counterstained with Gill-3 hematoxylin, mounted with Entellan, and examined and imaged under a light microscope. Prepared slides were evaluated and photographed using an Olympus BX50 light microscope. Detailed information regarding the primary antibodies used for Caspase-3, GluN2B, and TRPM2 immunostaining, including supplier, catalog number, working dilution, and RRID identifiers (where available), is provided in Table 5. Immunoreactivity was evaluated by an investigator blinded to the experimental group allocation. For each animal, five non-overlapping microscopic fields from comparable anatomical regions of the hippocampus and prefrontal cortex were examined at the same magnification. Staining intensity was assessed semi-quantitatively using ImageJ software (National Institutes of Health, Bethesda, MD, USA), and the mean optical density of the positively stained areas was calculated for each section. The average value obtained from all analyzed fields was used for statistical analysis. In addition, behavioral assessments (Open Field Test and Forced Swim Test), ELISA measurements, and qRT-PCR analyses were performed by investigators who were blinded to the experimental group allocation throughout data acquisition and analysis.

### 2.6 Statistical analyses

Statistical analyses were performed using IBM SPSS Statistics version 22.0 (IBM Corp., Armonk, NY, USA). Data are presented as mean ± standard deviation (SD). The normality of data distribution was assessed using the Shapiro–Wilk test, and homogeneity of variances was evaluated using Levene’s test. Differences among experimental groups were analyzed using one-way analysis of variance (ANOVA), followed by Tukey’s *post hoc* test for multiple pairwise comparisons when appropriate. A p value < 0.05 was considered statistically significant. For gene expression analyses involving multiple molecular targets, p values were adjusted using the Benjamini–Hochberg false discovery rate (FDR) procedure to reduce the likelihood of type I error. Because the behavioral tests and serum neurotransmitter measurements were predefined primary outcome measures involving a limited number of planned comparisons, no additional multiple-comparison adjustment was applied to these analyses. For the gene expression analyses, adjusted p values (q values) <0.05 were considered statistically significant.

## 3 Results

### 3.1 Assessment of body weight changes

**FIGURE 2:**
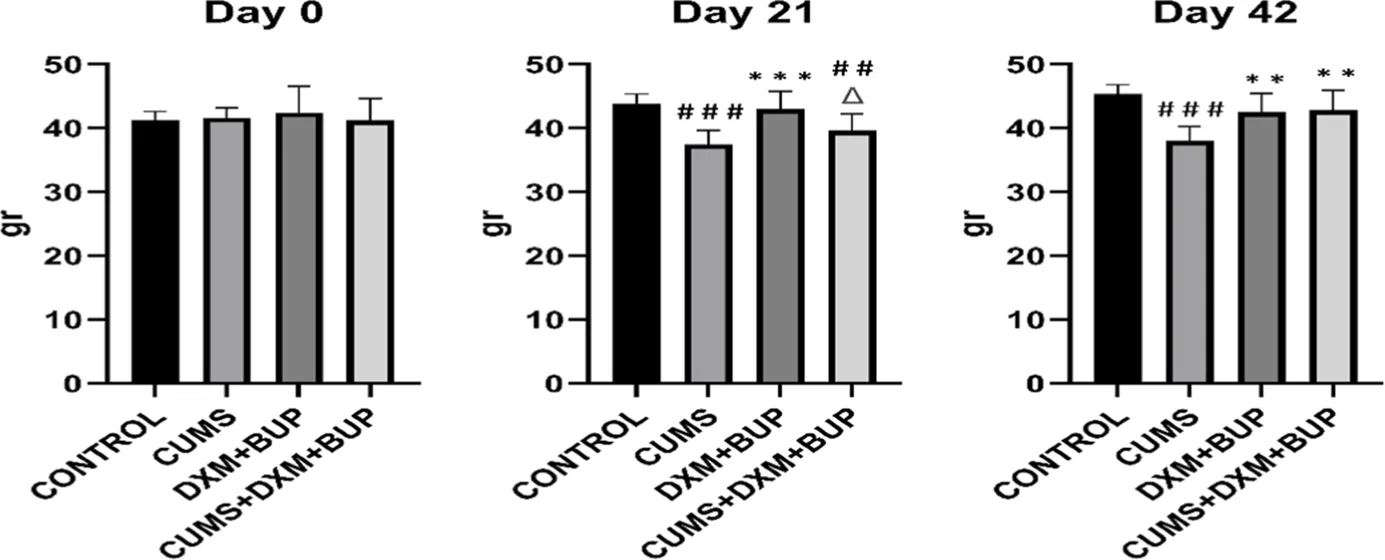
Changes in body weight of the groups during CUMS treatment. Data are shown as mean ± standard deviation. ^#^: Statistically significant change compared to the control group. *Statistically significant change compared to the CUMS group. Δ: Statistically significant change compared to the DXM + BUP group.

Repeated-measures ANOVA demonstrated a significant main effect of time (F = 3.88, p = 0.026) together with a significant time × group interaction (F = 9.26, p < 0.001). Baseline body weight values obtained on Day 0 were comparable across all experimental groups (p = 0.831). By day 21, a pronounced reduction in body weight was evident in the CUMS group, with pairwise comparisons confirming differences versus Control (p < 0.001), DXM + BUP (p < 0.001), and CUMS + DXM + BUP (p = 0.001). At this time point, animals in the CUMS + DXM + BUP group also exhibited lower body weight relative to Control (p = 0.006), whereas Control and DXM + BUP displayed overlapping distributions (p > 0.05). Evaluation of Day 42 measurements revealed persistent weight loss in CUMS animals, with significant reductions relative to Control (p < 0.001), DXM + BUP (p < 0.001), and CUMS + DXM + BUP (p < 0.001). Combined treatment under CUMS conditions was also associated with lower body weight compared with Control (p = 0.003), while Control and DXM + BUP groups remained comparable (p > 0.05) (Figure 2).

### 3.2 Assessment of sucrose preference

**FIGURE 3:**
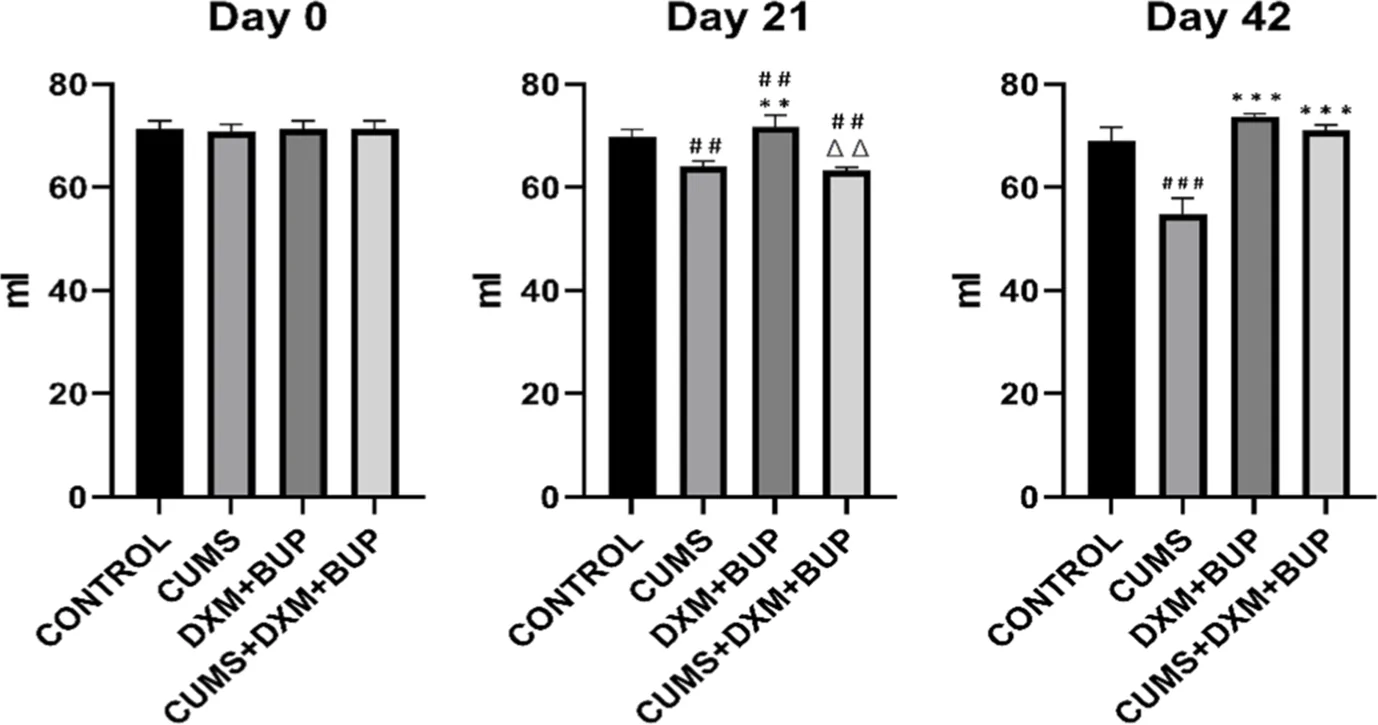
Changes in Sucrose preference of the groups. Data are shown as mean ± standard deviation. ^#^: Statistically significant change compared to the control group. *Statistically significant change compared to the CUMS group. Δ: Statistically significant change compared to the DXM + BUP group.

Repeated measures ANOVA results showed that the time factor had a significant effect (F = 25.30, p < 0.001). The time × group interaction was also found to be statistically significant (F = 25.67, p < 0.001). According to the one-way ANOVA results, no significant difference was found between the groups in basal sucrose preference measurements (p = 0.931). Post-hoc analyses showed that the CUMS group’s sucrose preference percentage was significantly lower than the Control (p = 0.007), DXM + BUP (p < 0.001), and CUMS + DXM + BUP (p = 0.004) groups. However, no significant difference was found between the Control and DXM + BUP groups (p > 0.05). The CUMS + DXM + BUP group’s sucrose preference also did not reach a statistically significant level compared to the Control group (p < 0.05). During the STT assessment on Day21, sucrose intake in the Control group was higher than in both the CUMS (p = 0.007) and CUMS + DXM + BUP (p = 0.004) groups. Comparable sucrose consumption was observed between the Control and DXM + BUP groups (p = 0.414). In contrast, CUMS animals exhibited lower sucrose intake than the DXM + BUP group (p = 0.001). Overlapping values were detected between the CUMS and CUMS + DXM + BUP groups (p = 0.946). Additionally, sucrose consumption in the DXM + BUP group exceeded that of the CUMS + DXM + BUP group (p = 0.001). Final measurements revealed a substantial decline in sucrose consumption in CUMS mice, with significant differences observed relative to Control (p < 0.001), DXM + BUP (p < 0.001), and CUMS + DXM + BUP (p < 0.001). CUMS + DXM + BUP treatment also demonstrated reduced sucrose preference compared with Control (p < 0.001). In contrast, Control and DXM + BUP groups displayed similar response profiles (p > 0.05) (Figure 3).

### 3.3 Behavioral test results

#### 3.3.1 Assessment of open field test performance

**FIGURE 4:**
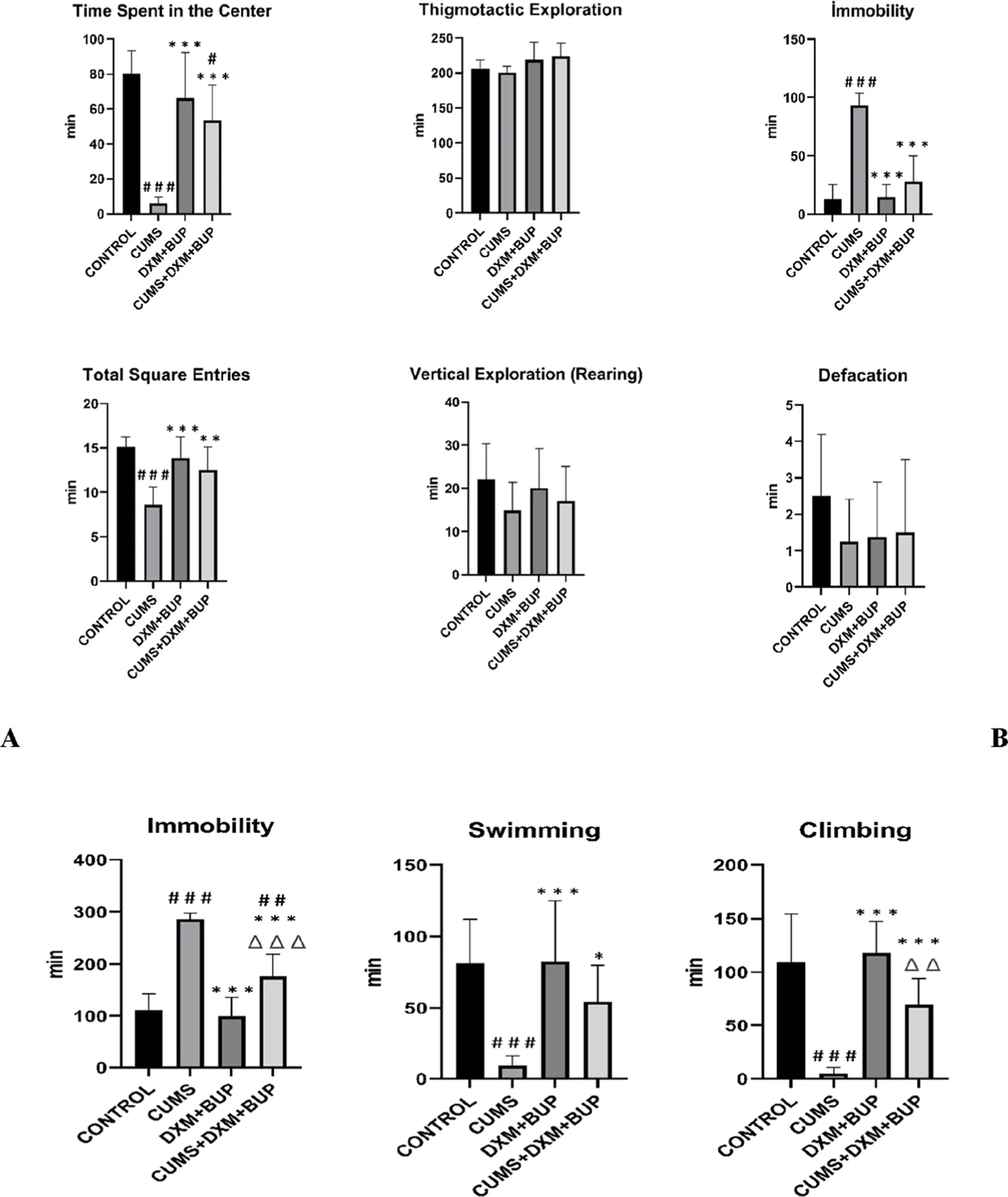
**(A)** Behavioral changes of the groups in OFT. **(B)** Behavioral changes of the groups in FST. Data are presented as mean ± standard deviation. ^#^: Statistically significant change compared to the control group. *: Statistically significant change compared to the CUMS group. Δ: Statistically significant change compared to the DXM + BUPgroup.

Time spent in the center zone was significantly reduced in the CUMS group compared with the Control (p < 0.001), DXM + BUP (p < 0.001), and CUMS + DXM + BUP (p < 0.001) groups. Control mice showed greater center activity than CUMS + DXM + BUP (p = 0.029). Control and DXM + BUP exhibited overlapping values (p > 0.05), as did DXM + BUP and CUMS + DXM + BUP (p > 0.05). Peripheral zone duration remained similar across groups (p > 0.05). Immobility duration increased prominently in the CUMS group relative to Control (p < 0.001), DXM + BUP (p < 0.001), and CUMS + DXM + BUP (p < 0.001). Comparable immobility values were observed between CUMS + DXM + BUP and both Control and DXM + BUP (p > 0.05), with Control and DXM + BUP also showing overlapping distributions (p > 0.05). Locomotor activity, assessed by total square crossings, was reduced in CUMS mice compared with Control (p < 0.001), DXM + BUP (p < 0.001), and CUMS + DXM + BUP (p = 0.005), whereas Control, DXM + BUP, and CUMS + DXM + BUP demonstrated similar activity levels (p > 0.05). Rearing and defecation frequencies did not vary among groups (p > 0.05) (Figure 4A).

#### 3.3.2 Assessment of forced swim test performance

CUMS exposure resulted in pronounced increases in immobility relative to Control (p < 0.001), DXM + BUP (p < 0.001), and CUMS + DXM + BUP (p < 0.001). Combined treatment under stress conditions partially attenuated this effect, although immobility remained elevated compared with Control (p = 0.003). Control and DXM + BUP exhibited overlapping immobility values (p > 0.05). In addition, immobility in the DXM + BUP group was lower than in CUMS + DXM + BUP (p < 0.001). Swimming behavior was reduced in CUMS mice compared with Control (p < 0.001), DXM + BUP (p < 0.001), and CUMS + DXM + BUP (p = 0.022). Control and DXM + BUP showed similar swimming times (p > 0.05). Although CUMS + DXM + BUP animals displayed lower swimming activity than Control and DXM + BUP, these differences did not reach significance (p > 0.05). Climbing activity followed a comparable pattern, with CUMS mice exhibiting lower values than Control (p < 0.001), DXM + BUP (p < 0.001), and CUMS + DXM + BUP (p = 0.001). DXM + BUP and Control remained aligned (p > 0.05), and overlapping climbing values were observed across Control, DXM + BUP, and CUMS + DXM + BUP (p > 0.05). By contrast, DXM + BUP produced higher climbing scores than CUMS + DXM + BUP (p = 0.015) (Figure 4B).

### 3.4 ELISA results

**FIGURE 5:**
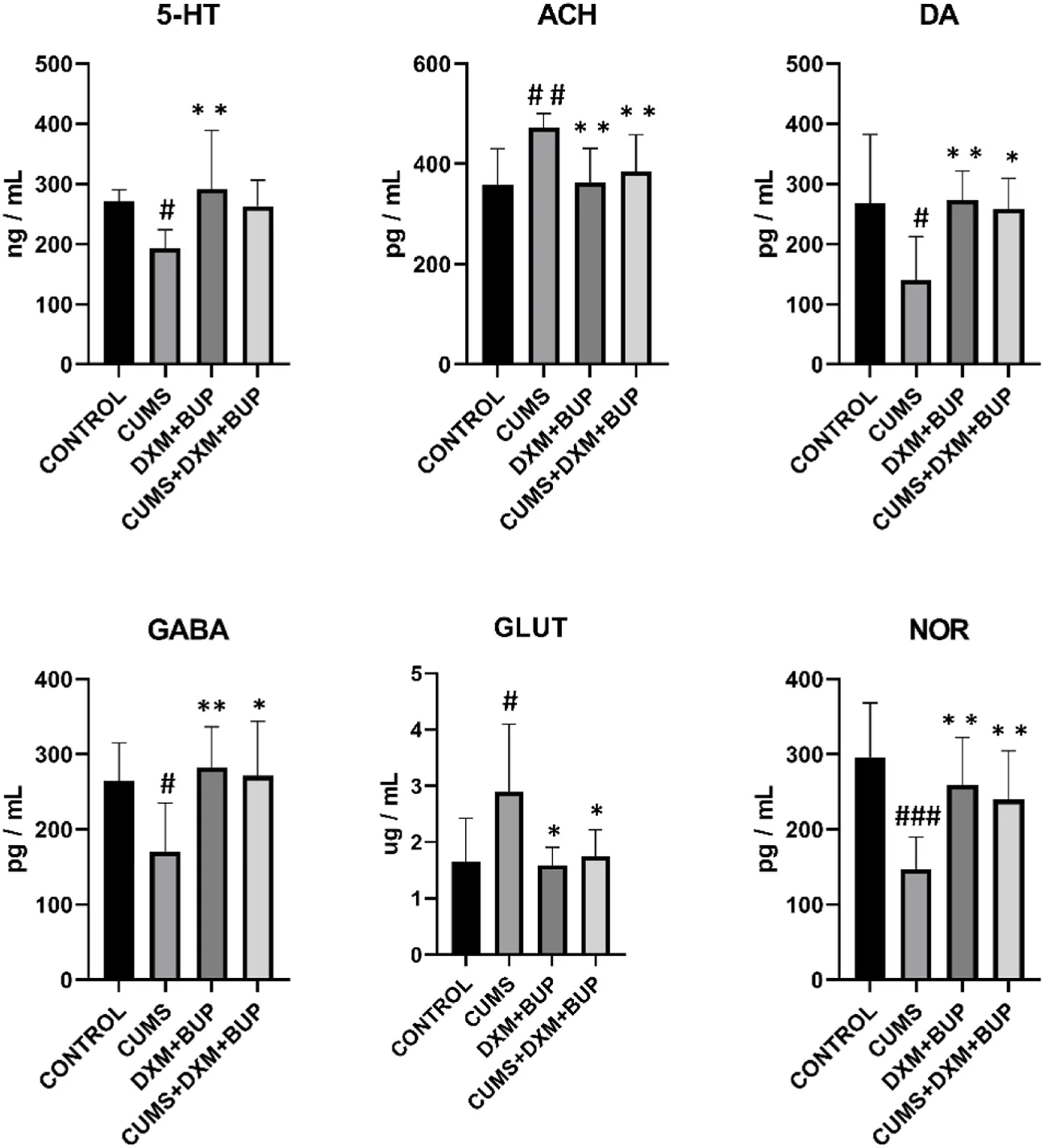
Changes in neurotransmitter levels of the groups. Data are shown as mean ± standard deviation. ^#^: Statistically significant change compared to the control group. *: Statistically significant change compared to the CUMS group. Δ: Statistically significant change compared to the DXM + BUP group.

One-way ANOVA revealed significant group differences in serotonin (F (3,28) = 4.632, p = 0.009), acetylcholine (F (3,28) = 5.636, p = 0.004), dopamine (F (3,28) = 5.537, p = 0.004), GABA (F (3,28) = 3.737, p = 0.022), glutamate (F (3,28) = 5.269, p = 0.005), and noradrenaline (F (3,28) = 5.810, p = 0.003). Detailed Tukey *post hoc* comparisons are summarized in Table 3. Post hoc Tukey analysis showed that serotonin concentrations were reduced in the CUMS group relative to the Control (p = 0.044) and DXM + BUP (p = 0.008) groups, while the CUMS + DXM + BUP group displayed values comparable to those of the remaining groups (p > 0.05). Acetylcholine levels were significantly increased in CUMS mice compared with the Control (p = 0.007), DXM + BUP (p = 0.008), and CUMS + DXM + BUP (p = 0.047) groups, whereas no significant differences were observed among the Control, DXM + BUP, and CUMS + DXM + BUP groups (p > 0.05). Dopamine levels were significantly lower in the CUMS group than in the Control (p = 0.012), DXM + BUP (p = 0.008), and CUMS + DXM + BUP (p = 0.022) groups, while these latter three groups did not differ significantly (p > 0.05). Similarly, GABA concentrations were reduced in the CUMS group compared with the Control (p = 0.033) and DXM + BUP (p = 0.038) groups, whereas no significant differences were detected among the remaining pairwise comparisons (p > 0.05). Glutamate levels were significantly elevated following CUMS exposure compared with the Control (p = 0.015), DXM + BUP (p = 0.010), and CUMS + DXM + BUP (p = 0.026) groups, while the latter three groups showed comparable values (p > 0.05). Finally, norepinephrine concentrations were significantly higher in the CUMS group than in the Control (p = 0.004) and DXM + BUP (p = 0.009) groups, whereas the remaining comparisons were not statistically significant (p > 0.05) (Figure 5).

**TABLE 3:** One-way ANOVA and Tukey post hoc analyses for behavioral tests and neurotransmitter measurements.

Variable	F (df = 3,28)	p	Significant tukey comparisons
Serotonin	4.632	0.009	Control vs. CUMS (0.044); AB vs. CUMS (0.008)
ACh	5.636	0.004	Control vs. CUMS (0.007); AB vs. CUMS (0.008); CUMS + AB vs. CUMS (0.047)
DA	5.537	0.004	Control vs. CUMS (0.012); AB vs. CUMS (0.008); CUMS + AB vs. CUMS (0.022)
GABA	3.737	0.022	Control vs. CUMS (0.033); AB vs. CUMS (0.038)
Glutamate	5.269	0.005	Control vs. CUMS (0.015); AB vs. CUMS (0.010); CUMS + AB vs. CUMS (0.026)
Noradrenaline	5.810	0.003	Control vs. CUMS (0.004); AB vs. CUMS (0.009)

### 3.5 qRT-PCR results

**FIGURE 6:**
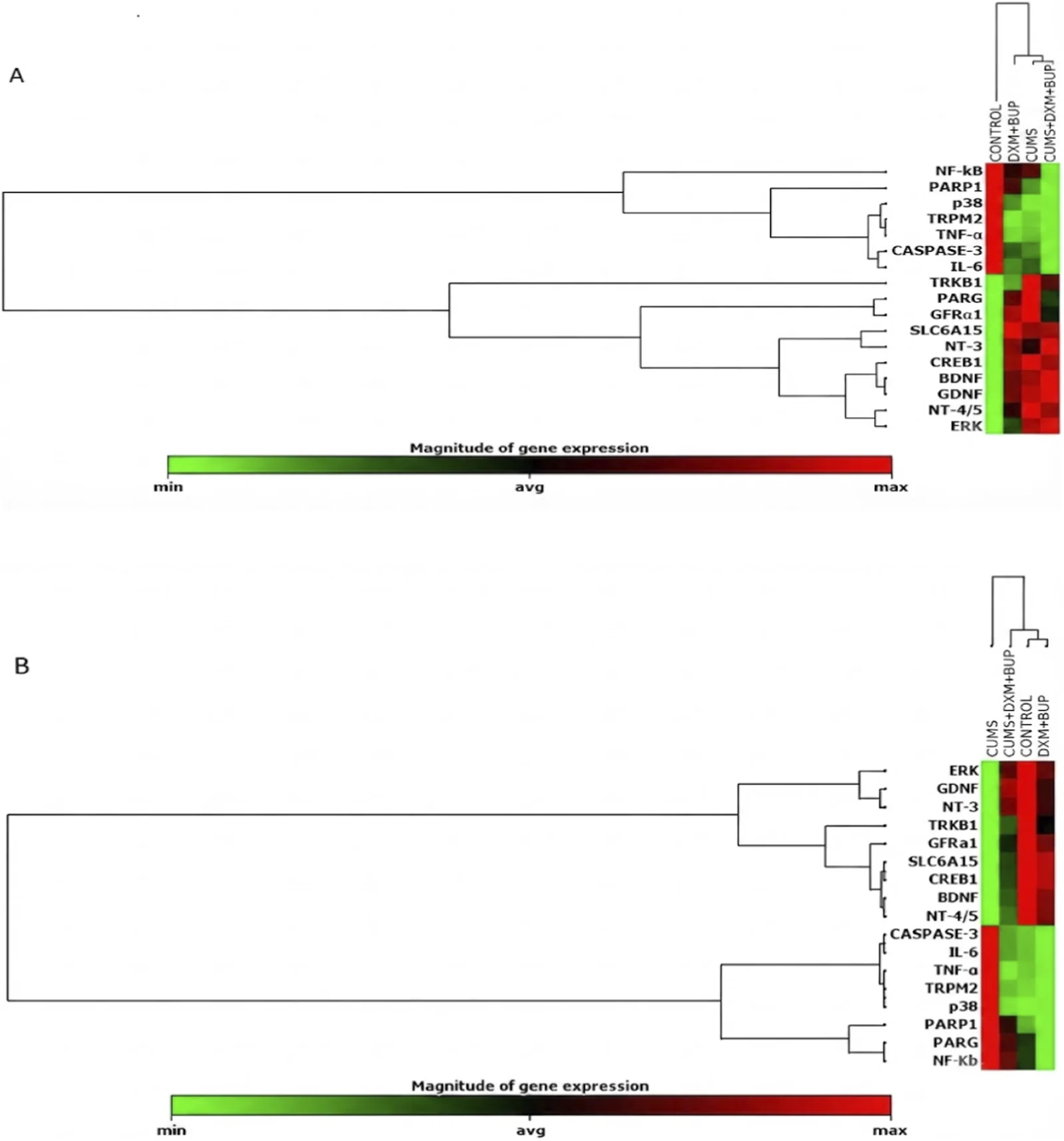
**(A)** Heatmap graph of gene expressions in the hippocampal tissue of Control, CUMS, DXM + BUP and CUMS + DXM + BUP groups. **(B)** Heatmap graph of gene expressions in the prefrotal cortex tissue of Control, CUMS, DXM + BUP and CUMS + DXM + BUP groups. The scale bar shows that the color varies from black to green or red according to the magnitude of gene expression.

In hippocampal tissue, compared with the Control group, the CUMS group exhibited significant downregulation of *Slc6a15* (p = 0.015), *Bdnf* (p = 0.019), *Trkb* (p = 0.025), *Ntf3* (p = 0.019), *Creb1* (p = 0.021), and *Erk* (p = 0.023), accompanied by increased expression of *Trpm2* (p = 0.019), *Casp3* (p = 0.013), *p38* (p = 0.033), *Tnf* (p = 0.040), and *Il6* (p = 0.032). Expression levels of these genes in the DXM + BUP group were comparable with those of the Control group (p > 0.05). Similarly, the CUMS + DXM + BUP group showed expression profiles approaching those of the Control group, with no statistically significant differences detected between these groups (p > 0.05). Compared with the CUMS group, DXM + BUP administration significantly increased the expression of *Slc6a15* (p = 0.013), *Bdnf* (p = 0.017), *Trkb* (p = 0.037), *Ntf3* (p = 0.010), *Creb1* (p = 0.027), and *Erk* (p = 0.022), while significantly reducing the expression of *Trpm2* (p = 0.015), *Casp3* (p = 0.006), *p38* (p = 0.029), *Tnf* (p = 0.030), and *Il6* (p = 0.011). Similarly, compared with the CUMS group, the CUMS + DXM + BUP group demonstrated increased expression of neuroplasticity-related genes, including *Slc6a15*, *Bdnf*, *Ntf3*, and *Creb1*, together with decreased expression of *Trpm2*, *Casp3*, *p38*, *Tnf*, and *Il6* (all p < 0.05). Expression profiles between the DXM + BUP and CUMS + DXM + BUP groups were comparable (p > 0.05) (Figure 6A). In the prefrontal cortex, CUMS exposure resulted in significant reductions in the expression of *Slc6a15*, *Bdnf*, *Gfra1*, *Ntf4*, and *Creb1* compared with the Control group (all p < 0.05). Conversely, expression levels of oxidative stress-, apoptotic-, and inflammatory pathway-related genes, including *Trpm2*, *Casp3*, *p38*, *Tnf*, and *Il6*, were significantly increased following CUMS exposure (all p < 0.05). The DXM + BUP group exhibited gene expression patterns comparable with those of the Control group (p > 0.05). In addition, compared with the CUMS group, DXM + BUP treatment significantly restored the expression of neurotrophic and signaling-related genes, including *Slc6a15*, *Bdnf*, *Ntf4*, and *Creb1*, while reducing the expression of *Trpm2*, *Casp3*, *p38*, *Tnf*, and *Il6* (all p < 0.05). The CUMS + DXM + BUP group demonstrated similar regulatory effects, with increased expression of neurotrophic-related genes and decreased expression of oxidative stress, apoptotic, and inflammatory markers compared with the CUMS group (all p < 0.05). Expression levels between the DXM + BUP and CUMS + DXM + BUP groups did not significantly differ (p > 0.05) (Figure 6B).

### 3.6 Immunohistochemical results

**FIGURE 7:**
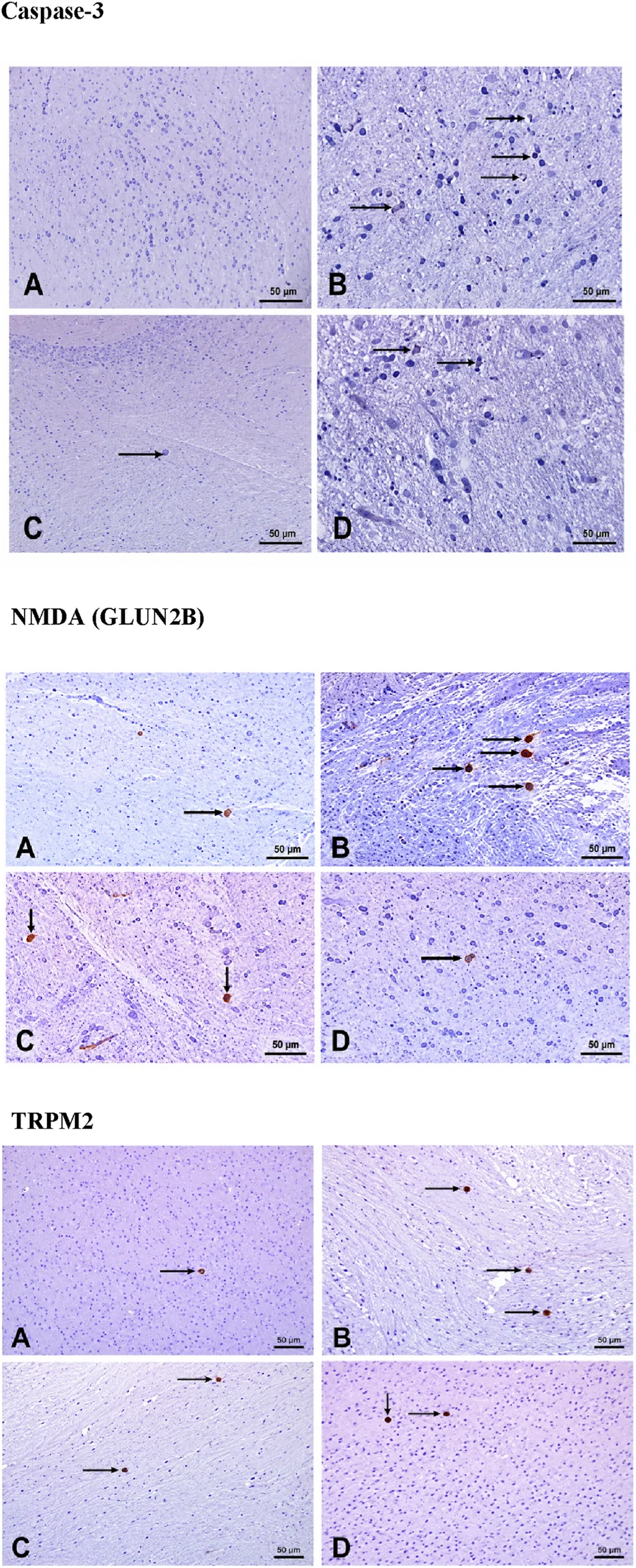
The immunohistochemistry staining of Caspase-3, NMDA (GLUN2B) and, TRPM2 in brain slices. **(A)** Control, **(B)** CUMS, **(C)** DXM + BUP, **(D)** CUMS + DXM + BUP.

**FIGURE 8:**
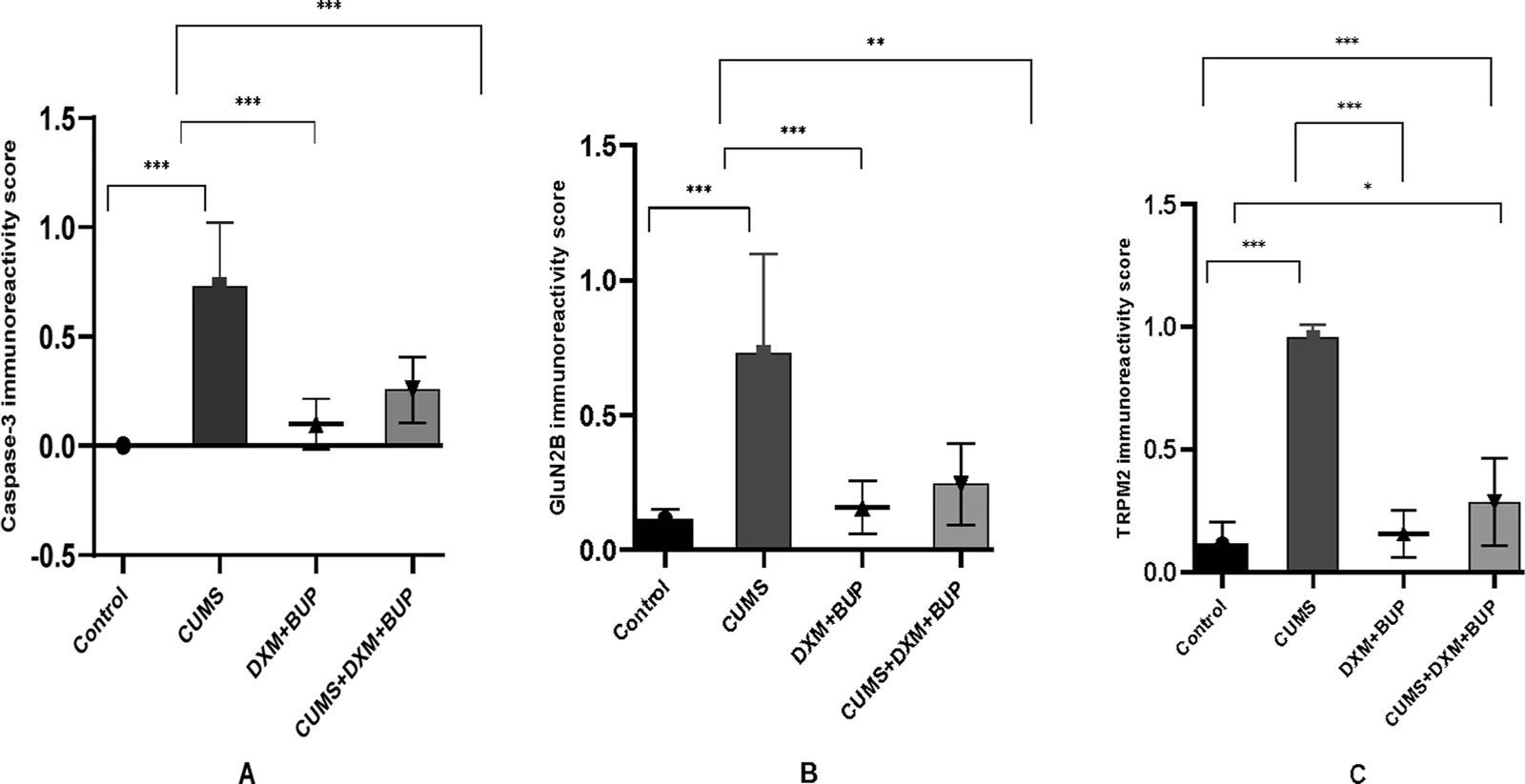
Quantitative immunohistochemical analysis of **(A)** Caspase-3, **(B)** GluN2B, and **(C)** TRPM2 immunoreactivity in the Control, CUMS, DXM + BUP, and CUMS + DXM + BUP groups. Data are presented as mean ± SD (n = 7 per group). Statistical analyses were performed using one-way ANOVA followed by Tukey’s *post hoc* test. Significant differences are indicated as *p < 0.05, **p < 0.01, and ***p < 0.001.

One-way ANOVA demonstrated significant differences among the groups for Caspase-3 (F (3,24) = 23.906, p < 0.001), GluN2B (F (3,24) = 13.289, p < 0.001), and TRPM2 (F (3,24) = 83.119, p < 0.001). Detailed Tukey post hoc comparisons are presented in Table 4. Post hoc Tukey analysis showed that Caspase-3 immunoreactivity was significantly higher in the CUMS group than in the Control (p < 0.001), DXM + BUP (p < 0.001), and CUMS + DXM + BUP (p < 0.001) groups. No significant difference was observed between the Control and DXM + BUP groups (p = 0.710), or between the DXM + BUP and CUMS + DXM + BUP groups (p = 0.354), whereas the difference between the Control and CUMS + DXM + BUP groups was borderline significant (p = 0.050). Similarly, GluN2B immunoreactivity was significantly increased in the CUMS group compared with the Control (p < 0.001), DXM + BUP (p < 0.001), and CUMS + DXM + BUP (p = 0.001) groups. No significant differences were detected among the Control, DXM + BUP, and CUMS + DXM + BUP groups (all p > 0.05). TRPM2 immunoreactivity also differed significantly among the experimental groups. The CUMS group exhibited significantly higher TRPM2 staining than the Control (p < 0.001), DXM + BUP (p < 0.001), and CUMS + DXM + BUP (p < 0.001) groups. In addition, TRPM2 immunoreactivity remained slightly but significantly higher in the CUMS + DXM + BUP group than in the Control group (p = 0.044), whereas no significant differences were observed between the Control and DXM + BUP groups (p = 0.895) or between the DXM + BUP and CUMS + DXM + BUP groups (p = 0.178) (Figures 7, 8).

**TABLE 4:** One-way ANOVA and Tukey *post hoc* analyses of immunohistochemical markers.

Marker	F (3,24)	p value	Significant tukey comparisons
Caspase-3	23.906	<0.001	Control vs. CUMS (p < 0.001); AB vs. CUMS (p < 0.001); CUMS + AB vs. CUMS (p < 0.001)
GluN2B	13.289	<0.001	Control vs. CUMS (p < 0.001); AB vs. CUMS (p < 0.001); CUMS + AB vs. CUMS (p = 0.001)
TRPM2	83.119	<0.001	Control vs. CUMS (p < 0.001); AB vs. CUMS (p < 0.001); CUMS + AB vs. CUMS (p < 0.001)

**TABLE 5:** Antibodies used for immunohistochemical analysis.

Target protein	Primary antibody	Supplier	Catalog number	Dilution	RRID
Caspase-3	Affinity biosciences	AF6311	1:100	N/A	Caspase-3
TRPM2	Affinity biosciences	DF7533	1:100	N/A	TRPM2
NR2B (GluN2B)	Affinity biosciences	DF8580	1:100	N/A	NR2B (GluN2B)

## 4 Discussion

In this study, we characterized the behavioral, neurotransmitter-related, neurotrophic, inflammatory, and apoptosis-associated molecular alterations induced by CUMS and evaluated the therapeutic effects of the dextromethorphan–bupropion (DXM + BUP) combination on these stress-related impairments. The pronounced weight loss observed in the CUMS group is a frequently reported outcome in chronic stress paradigms and reflects the physiological sensitivity of the model (Willner, 2005). The attenuation of weight loss in the CUMS + DXM + BUP group compared with the CUMS group suggests that the treatment partially mitigated the stress-driven physiological response. In line with this interpretation, NMDAR inhibitors such as ketamine have been reported to alleviate stress-associated weight loss in CUMS models (Garcia et al., 2009). In addition, monoaminergic antidepressants have been shown to partially reverse weight loss in chronic stress models (Buran et al., 2017). Therefore, the reduced weight loss in the CUMS + DXM + BUP group may reflect a combined effect involving both a reduction in excitatory load via NMDA antagonism and an improvement in monoaminergic transmission.

In our study, reduced sucrose preference in the CUMS group reflects impaired reward sensitivity, which is considered one of the most characteristic indicators of anhedonia in chronic stress models (Willner, 1997). The increase in sucrose preference in the DXM + BUP group may be attributed to dextromethorphan-mediated modulation of glutamatergic transmission via sigma-1 receptor activation and NMDA receptor antagonism, thereby producing antidepressant-like effects, together with bupropion-mediated inhibition of dopamine and norepinephrine reuptake, which may enhance motivational processes and reward processing capacity (Stahl, 2004; Lauterbach, 2011; Nguyen et al., 2014). Reports indicating that NMDA antagonists such as ketamine and memantine reduce sucrose preference loss in chronic stress models further support a mechanistic concordance with the improvement in anhedonia observed here following DXM + BUP administration (Li et al., 2010; Reus et al., 2012).

In the present study behavioral assessments demonstrated that chronic stress markedly disrupted anxiety-related behavior and psychomotor activity, whereas the DXM + BUP combination reversed a substantial proportion of these impairments. In the open field test, reduced time spent in the center and decreased locomotor activity in CUMS-exposed animals are consistent with an anxiety-like profile and psychomotor slowing commonly reported in chronic stress models (Buran et al., 2022). Similarly, increased immobility time in the forced swim test indicates behavioral despair and motivational impairment in the CUMS group. The reduction of depressive-like behaviors in the CUMS + DXM + BUP group in both tests may be explained not only by dextromethorphan-induced modulation of glutamatergic signaling via NMDA antagonism but also by sigma-1 receptor activation, which can influence cellular processes related to Ca^2+^ homeostasis and endoplasmic reticulum–mitochondria communication under stress conditions (Hayashi, 2015; Stahl, 2019). Dextromethorphan has been shown to reduce immobility time in the FST, and this antidepressant-like effect can be attenuated by sigma-1 receptor antagonism (Nguyen, 2014). Evidence that sigma-1 receptor signaling can modulate BDNF-related neuroplasticity pathways (Fujimoto et al., 2012; Yagasaki et al., 2006), together with data indicating that NMDA receptor antagonists such as ketamine and memantine can reverse anhedonia and depressive-like behaviors in chronic stress models, supports the interpretation that the improvements observed in the OFT and FST are compatible with glutamatergic modulation and sigma-1–mediated cellular adaptation mechanisms (Li et al., 2010; Reus and Quevedo, 2012). This behavioral pattern is consistent with clinical findings demonstrating early symptom improvement with the dextromethorphan–bupropion combination (Iosifescu, 2022b; Tabuteau, 2022b).

Our ELISA findings indicate that CUMS induced marked disruptions in monoaminergic signaling and in the excitatory–inhibitory balance, whereas DXM + BUP treatment ameliorated these alterations through multimodal mechanisms. Across the CUMS literature, both concordant and divergent results have been reported for neurotransmitter levels, likely reflecting the pronounced biological heterogeneity of depression, differences in CUMS duration and intensity, and variability in experimental conditions (e.g., species, age, sampling time, and sample type) (Buran et al., 2022; Willner, 2017; Yankelevitch-Yahav et al., 2015). Several studies have shown that CUMS suppresses serotonergic activity in the hippocampus and prefrontal cortex tissue, accompanied by dopaminergic alterations (Lu et al., 2019). Chronic stress models have also been associated with reduced GABAergic tone and disrupted excitatory–inhibitory balance, with increased glutamatergic activity reported in some paradigms (Luscher and Fuchs, 2011; Yin J. et al., 2020). In this context, the decreases in 5-HT, DA, and GABA levels and the increase in glutamate levels observed in our CUMS group suggest that chronic stress exerts suppressive and destabilizing effects across multiple neurotransmitter systems. In the present study, the post-treatment recovery of 5-HT levels is compatible with the monoaminergic regulatory effects of bupropion and may also involve dextromethorphan-mediated contributions to neuroregulatory processes via sigma-1 receptor agonism and NMDA receptor antagonism (Stahl, 2013; Maurice and Su, 2009). Similarly, NMDA receptor antagonists have been reported to reverse depressive-like phenotypes in chronic stress models and to partially normalize stress-related neurochemical changes (Réus, 2012). Reduced dopamine levels in the CUMS group imply functional suppression within reward-related circuits. The normalization of dopamine levels following DXM + BUP treatment is consistent with bupropion’s inhibition of dopamine and norepinephrine reuptake and suggests that dextromethorphan, via sigma-1 receptor activation and NMDA antagonism, may indirectly support dopaminergic circuit function under stress (Stahl, 2013; Nguyen et al., 2014). Indeed, bupropion has been shown experimentally to significantly increase extracellular DA, NOR, and 5-HT levels in the rat hippocampus (Pani et al., 2003).

The reduction in GABA levels under CUMS supports the notion that stress suppresses inhibitory transmission and disrupts the glutamate–GABA cycle (Luscher and Fuchs, 2011; Yin X. et al., 2020). The restoration of GABA levels after treatment may reflect a combined contribution from dextromethorphan’s ability to constrain glutamatergic hyperactivity and bupropion’s monoaminergic modulation, which may indirectly support GABAergic transmission (Stahl, 2013). In our study, the increase in glutamate levels in the CUMS group parallels clinical findings regarding the glutamatergic component of depression (Yin J. et al., 2020; Wang et al., 2022). In CUMS-exposed animals, increased hippocampal glutamate has been reported to correlate with depressive-like behaviors and to amplify synaptic stress responses (Yin X. et al., 2020). Accordingly, the normalization of glutamate levels in the treatment groups suggests that the DXM + BUP combination may counterbalance pathological stress-related excitatory load. Excessive NMDA receptor activation and glutamate excitotoxicity are increasingly recognized as key contributors to MDD pathophysiology, and recent evidence suggests that glutamate excitotoxicity may be associated with overactivation of TRP channels (Zong et al., 2025). The decreased norepinephrine (NOR) levels observed in the CUMS group are consistent with experimental data indicating that chronic stress can suppress the noradrenergic system (Qiao et al., 2020). The reversal of this change by DXM + BUP can be explained primarily by bupropion’s strong norepinephrine–dopamine reuptake inhibitor (NDRI) properties, which inhibit synaptic NOR reuptake (Stahl, 2004; Pani et al., 2003). In addition, NMDA receptor blockade has been reported to modulate stress-related monoaminergic responses (Willner, 2017; Duman and Aghajanian, 2012). Taken together, these neurotransmitter normalizations support the biological basis of the observed behavioral improvement. In the present study, neurotransmitter concentrations were measured in serum rather than in brain tissue because serum analysis provides a standardized and minimally invasive approach that enables the simultaneous quantification of multiple neurotransmitters. Although region-specific measurements in the hippocampus or prefrontal cortex would provide more direct mechanistic information regarding central neurotransmission, peripheral neurotransmitter levels have been widely used as complementary biomarkers of systemic neurochemical alterations in experimental depression models. Therefore, the serum findings should be interpreted together with the behavioral, gene expression, and immunohistochemical results rather than as direct measures of neurotransmitter concentrations within specific brain regions.

The effects of CUMS in the brain are characterized by suppression of neurotrophin signaling and robust activation of an oxidative stress–driven inflammation–apoptosis axis (Willner, 2017; Duman and Aghajanian, 2012). In our study, CUMS produced a region-specific yet convergent molecular impairment profile in both the hippocampus and prefrontal cortex tissue. These findings suggest that chronic stress suppresses hippocampal BDNF, TrkB, CREB, and ERK signaling pathways, and that decreases in these factors may compromise synaptic plasticity processes that are critical for learning, memory, and emotional regulation (Jia et al., 2021; Numakawa and Kajihara, 2023). Decreases in SLC6A15 and NT-3 have been associated with increased stress vulnerability and heightened hippocampal stress reactivity (Kohli et al., 2011; Santarelli et al., 2017b). In the prefrontal cortex, reductions in NT-4/5 and GFRα1 in addition to decreases in neurotrophic genes indicate that a broader neurotrophin network supporting executive function, motivation, and decision-making is affected by stress. Several studies have reported that the prefrontal BDNF–TrkB–CREB axis is weakened under stress (Gourley et al., 2008; Gray et al., 2013) and that reduced ERK signaling is associated with loss of prefrontal function (Morris et al., 2014).

Another key finding of our study is the pronounced increase in the inflammation–apoptosis axis in both brain regions, with TRPM2 upregulation appearing central to this molecular response. TRPM2 is a ROS-sensitive Ca^2+^ channel activated via PARP1/ADPR signaling, and its activation can trigger p38–NF-κB pathways, thereby facilitating pro-inflammatory cytokine production and apoptotic processes (Jeon et al., 2012; Xie et al., 2018; Zeng and Wang, 2020). Several studies have reported a significant increase in p38, NF-κB, TNF-α, and IL-6 along with TRPM2 activation in CUMS models (Wang and Zhang, 2018; Zhang and Chen, 2021). In the hippocampus, increased TRPM2, p38, TNF-α, IL-6, and Caspase-3 likely enhance inflammatory burden within circuits mediating learning and emotional regulation, whereas similar increases in the prefrontal cortex suggest stress-related pressure on executive and motivational circuitry (Wang and Zhang, 2018; Liu and Sun, 2018). DXM + BUP treatment in our study strengthened the BDNF–TrkB–CREB–ERK axis suppressed by CUMS in both the hippocampus and prefrontal cortex tissue and promoted recovery of complementary neurotrophins such as NT-3 and NT-4/5, contributing to a more integrated reactivation of the plasticity network.

In addition, sigma-1 receptor agonism has been reported to modulate Ca^2+^ homeostasis and CREB-mediated transcriptional processes, providing further biological rationale for the neurotrophic increases observed here (Nguyen et al., 2014). In our study, the partial restoration of SLC6A15 expression by DXM + BUP suggests that the combination may improve not only neurotrophic responses but also stress-related regulation of metabolic/transport systems (Kohli et al., 2011; Zhang et al., 2015; Santarelli et al., 2015). Moreover, DXM + BUP reduced TRPM2, p38, TNF-α, IL-6, and Caspase-3 levels, indicating suppression of the ROS-sensitive inflammatory response. Parallel pharmacodynamic effects have been reported, including ketamine-mediated reductions in p38 and NF-κB activation with consequent decreases in TNF-α and IL-6 synthesis in the CUMS model (Réus, 2010), and bupropion-associated reductions in pro-inflammatory cytokines (Hwang and Lee, 2008). It is plausible that dextromethorphan-mediated NMDA antagonism limits Ca^2+^ overload, thereby indirectly attenuating PARP1–ADPR–dependent TRPM2 activation and weakening downstream p38/NF-κB signaling (Jeon et al., 2012; Zeng and Wang, 2020). Collectively, our findings indicate that a combination therapy simultaneously targeting neurotrophic insufficiency and TRPM2-mediated inflammatory burden can elicit broad-spectrum molecular recovery in the CUMS model.

In this study, immunohistochemical analyses demonstrated marked increases in TRPM2, NMDA, and Caspase-3 immunoreactivity in the CUMS group, supporting the RT-PCR findings of elevated TRPM2, p38, TNF-α, IL-6, and Caspase-3 and indicating robust activation of the inflammation–apoptosis axis under stress. Increased NMDA receptor positivity in the CUMS group is consistent with evidence of heightened glutamatergic load under chronic stress. The reductions in TRPM2, NMDA, and Caspase-3 levels in the CUMS + DXM + BUP group compared with the CUMS group suggest that the combination therapy alleviated inflammatory burden and excitatory hyperexcitability. These findings are consistent with the NMDA receptor antagonism of dextromethorphan, which may reduce glutamatergic excitotoxicity and downstream TRPM2-mediated inflammatory signaling (Li et al., 2010; Reus et al., 2010). When considered together with bupropion’s monoaminergic regulatory effects, these mechanisms may explain the broad-spectrum recovery observed across the inflammation–apoptosis axis (Stahl et al., 2013). Nevertheless, while our RT-PCR findings indicated pronounced suppression with treatment, only partial improvement was observed at the protein level, suggesting that restoration at the protein level may occur more slowly than transcriptional recovery. Taken together, these results indicate that DXM + BUP treatment mitigates CUMS-induced molecular damage by reducing the TRPM2-mediated inflammation–apoptosis axis and NMDA-mediated excitatory load, although delayed recovery may persist for certain protein-level alterations.

### 4.1 Limitations

An important limitation of the present study is that only male mice were included. Because sex differences are known to influence susceptibility to chronic stress, neuroinflammatory responses, neuroplasticity, and antidepressant efficacy, the present findings cannot be directly generalized to female animals. Future studies including both sexes are warranted to determine whether the therapeutic effects of the dextromethorphan–bupropion combination differ according to sex. Additionally, the study evaluated only a single dose and treatment duration of the dextromethorphan–bupropion combination; therefore, dose–response relationships and long-term effects remain to be determined. Although behavioral, neurochemical, gene expression, and immunohistochemical analyses were performed, direct mechanistic validation of the identified molecular pathways was not conducted. Furthermore, while the CUMS model is a widely used experimental model for investigating depression-related behaviors, it cannot fully reproduce the complex clinical heterogeneity of human depression. Food and water intake were not recorded throughout the CUMS protocol. Although body weight was monitored as an indicator of the physiological impact of chronic stress, future studies should include systematic monitoring of food and water consumption to provide additional evidence for model validation and treatment effects. Finally, although multiple-comparison correction was applied to the gene expression analyses, these molecular findings should be considered exploratory and require independent validation in future studies. Future investigations using different doses, longer follow-up periods, both sexes, complementary mechanistic approaches, and independent validation cohorts are needed to further clarify the therapeutic potential of the dextromethorphan–bupropion combination.

## 5 Conclusion

This study comprehensively demonstrated the behavioral, neurochemical, and molecular disruptions induced by CUMS and showed that the dextromethorphan–bupropion combination can markedly improve this multi-level stress response. Treatment reduced anhedonia and behavioral despair and rebalanced neurotransmitter homeostasis. Reactivation of the suppressed BDNF–TrkB–CREB–ERK axis in the hippocampus and prefrontal cortex tissue, together with a reduction in inflammatory burden along the TRPM2–p38–NF-κB–TNF-α/IL-6 pathway, indicates concomitant recovery of plasticity and cellular integrity. The immunohistochemical decreases in TRPM2, GLUN2B, and Caspase-3 further supported this molecular improvement. Overall, our findings suggest that the DXM + BUP combination may represent an effective therapeutic strategy that simultaneously targets neurotrophic insufficiency and the TRPM2-mediated inflammation–apoptosis axis to alleviate CUMS-induced depressive phenotypes. These findings support further investigation of the DXM + BUP combination as a potential therapeutic strategy for stress-related depression and possibly treatment-resistant depression.

## Data Availability

The original contributions presented in the study are included in the article/supplementary material, further inquiries can be directed to the corresponding author.
